# Characterization of the chloroplast genome of the family Lauraceae plant species, *Cinnamomum cassia*

**DOI:** 10.1080/23802359.2019.1687360

**Published:** 2019-11-08

**Authors:** Kai Song, Man He, Jingbo Yu, Yanjun Guan, Yumeng Bai, Shuquan Xin, Tianyi Cao

**Affiliations:** a School of Life Science, Changchun Normal University, Changchun, China;; b Zhejiang Chinese Medical University, Hangzhou, China

**Keywords:** *Cinnamomum cassia*, Lauraceae, chloroplast genome, evolutionary relationship

## Abstract

The fruit of *Cinnamomum cassia* is an important spice material and its branch is a common Chinese herbal medicine as the family Lauraceae. In this study, we reported the complete chloroplast genome of *C. cassia.* The chloroplast genome of *C. cassia* with length of 152,675 bp is a characteristic quadripartite structure. The length of the inverted-repeats regions (IRs), large single-copy (LSC) region, and small single-copy (SSC) region of *C. cassia* was 20,068 bp, 93,663 and 18,876 bp. The chloroplast genome of *C. cassia* contains 124 genes, which includes 80 protein-coding genes (PCGs), 36 transfer RNA genes (tRNAs) and 8 ribosomal RNA genes (rRNAs). The overall nucleotide content of the chloroplast genome: 30.0% A (Adenine), 30.8% T (Thymine), 19.7% C (Cytosine), 19.5% G (Guanine), and 39.2% GC content. Evolutionary relationship result showed that *Cinnamomum cassia* was most closely related to *Cinnamomum parthenoxylon* in the family Lauraceae by the Neighbor-Joining (NJ) method.

*Cinnamomum cassia* is named Gui-Zhi in Chinese as one of Chinese herbal medicine. It belongs to the genus Cinnamomum family Lauraceae that Originated in China. It is mainly with the aroma and essence function, which can be incorporated into different varieties of foodstuffs, perfumes and medicinal products (Huang et al. 2007). Medicinal use of *Cinnamomum cassia* dates back approximately 5000 years, when it was primarily used for the treatment of diarrhea, upset stomach and bad breath, as well as for relief of poor appetite, nausea and cramps (Hoehn and Stockert 2012). At present, the research of *C. cassia* mainly focuses on the medicinal ingredients. So, we want to know more about the genetic data of *C. cassia.* In our study, the chloroplast genome of *C. cassia* was reported, which can fill in the gaps of the chloroplast genome information, also for can provide genome data and information of the family Lauraceae species in further.

The fresh branches sample of *Cinnamomum cassia* was collected from Zhejiang Chinese Medical University (Hangzhou, Zhejiang, China, 30.09 N, 119.89E). The chloroplast DNA of *C. cassia* was extracted from the fresh branches using the modified CTAB method and stored in Zhejiang Chinese Medical University (No. SCMC-ZJU-TCM-03). The chloroplast DNA was purified and fragmented using the NEB Next Ultra^TM^ II DNA Library Prep Kit (NEB, BJ, and CN) that was sequenced and analyzed. FastQC software (Andrews 2015) was used to perform and remove low-quality reads and adapters for quality control. The chloroplast genome was assembled and annotated using the MitoZ software (Meng et al. 2019). OrganellarGenomeDRAW web (Greiner et al. 2019) was used to draw the physical map of the chloroplast genome of *C. cassia*.

The chloroplast genome of *Cinnamomum cassia* (NCBI accession No.AP5433123) with length of 152,675 bp is a characteristic quadripartite structure. The length of the inverted-repeats regions (IRs), large single-copy (LSC) region, and small single-copy (SSC) region of *C. cassia* was 20,068 bp, 93,663 and 18,876 bp. The chloroplast genome of *C. cassia* contains 124 genes, which includes 80 protein-coding genes (PCGs), 36 transfer RNA genes (tRNAs) and 8 ribosomal RNA genes (rRNAs). Total of 15 genes were found every IR region, including 5 PCG genes species (*ycf2 ,ndhB, rps7, rps12 a*nd *ycf1*), 6 tRNA genes species (*trnL-CAA, trnV-GAC, trnI-GAU, trnI-GAU, trnR-ACG* and *trnN-GUU*) and 4 rRNA genes species (*rrn16, rrn23, rrn4.5* and *rrn5*). The the overall nucleotide content of the chloroplast genome: 30.0% A (Adenine), 30.8% T (Thymine), 19.7% C (Cytosine), 19.5% G (Guanine), and 39.2% GC content.

Furthermore, based on 12 published chloroplast genome sequences with *C. cassia* to study the phylogenetic position. Neighbor-Joining (NJ) phylogenetic tree used the NJ method by MEGA X (Kumar et al. 2018) and performed using 2,000 bootstrap values replicate at each node. The final NJ phylogenetic tree was edited using the iTOL version 4.0 (https://itol.embl.de/) (Letunic and Bork 2016). The reconstructed phylogenetic tree result (Figure 1) showed that *Cinnamomum cassia* was most closely related to *Cinnamomum parthenoxylon* (MH050971.1) in evolutionary relationship. In the study, the chloroplast genome of *C. cassia* will be very important for genome data and information of the family Lauraceae species in further.

**Figure 1.**
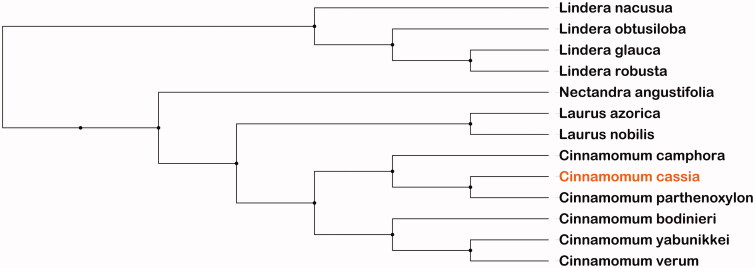
Phylogenetic relationships for *Cinnamomum cassia* based on other 12 species chloroplast genome sequences by the Neighbor-Joining (NJ) method analysis. Bootstrap support values based on 2,000 replicates are shown next to the nodes for each branch. These chloroplast genome sequences NCBI accession number as：*Cinnamomum parthenoxylon MH050971.1, Cinnamomum camphora NC_035882.1, Cinnamomum yabunikkei NC_044864.1, Cinnamomum verum NC_035236.1, Cinnamomum bodinieri MH394416.1, Laurus azorica MK041220.1, Laurus nobilis NC_034700.1, Lindera glauca NC_035953.1, Lindera robusta MH220738.1, Lindera obtusiloba MH220737.1, Lindera nacusua MH220736.1, Nectandra angustifolia MF939340.1.*
